# National health accounts data from 1996 to 2010: a systematic review

**DOI:** 10.2471/BLT.14.145235

**Published:** 2015-05-15

**Authors:** Anthony L Bui, Rouselle F Lavado, Elizabeth K Johnson, Benjamin PC Brooks, Michael K Freeman, Casey M Graves, Annie Haakenstad, Benjamin Shoemaker, Michael Hanlon, Joseph L Dieleman

**Affiliations:** a Institute for Health Metrics and Evaluation, University of Washington, 2301 5th Avenue, Suite 600, Seattle, WA 98121, United States of America (USA).; b The World Bank, Washington, USA.; c Information School, University of Washington, Seattle, USA.; d Indeed Corporation, Austin, USA.

## Abstract

**Objective:**

To collect, compile and evaluate publicly available national health accounts (NHA) reports produced worldwide between 1996 and 2010.

**Methods:**

We downloaded country-generated NHA reports from the World Health Organization global health expenditure database and the Organisation for Economic Co-operation and Development (OECD) StatExtract website. We also obtained reports from Abt Associates, through contacts in individual countries and through an online search. We compiled data in the four main types used in these reports: (i) financing source; (ii) financing agent; (iii) health function; and (iv) health provider. We combined and adjusted data to conform with OECD’s first edition of *A system of health accounts manual,* (2000).

**Findings:**

We identified 872 NHA reports from 117 countries containing a total of 2936 matrices for the four data types. Most countries did not provide complete health expenditure data: only 252 of the 872 reports contained data in all four types. Thirty-eight countries reported an average not-specified-by-kind value greater than 20% for all data types and years. Some countries reported substantial year-on-year changes in both the level and composition of health expenditure that were probably produced by data-generation processes. All study data are publicly available at http://vizhub.healthdata.org/nha/.

**Conclusion:**

Data from NHA reports on health expenditure are often incomplete and, in some cases, of questionable quality. Better data would help finance ministries allocate resources to health systems, assist health ministries in allocating capital within the health sector and enable researchers to make accurate comparisons between health systems.

## Résumé

**Objectif:**

Collecter, réunir et évaluer les rapports publics sur les comptes nationaux de la santé (CNS) produits à travers le monde entre 1996 et 2010.

**Méthodes:**

Nous avons téléchargé les rapports sur les CNS produits par différents pays à partir de la base de données sur les dépenses de santé dans le monde de l'Organisation mondiale de la Santé et du site Internet StatExtracts de l'Organisation de Coopération et de Développement Économiques (OCDE). Nous nous sommes également procurés des rapports auprès d'Abt Associates grâce à des contacts dans différents pays et à des recherches en ligne. Nous avons consolidé les quatre principaux types de données utilisés dans ces rapports: (i) source de financement; (ii) agent de financement; (iii) fonction de santé; et (iv) prestataire de santé. Nous avons combiné et ajusté les données de façon à respecter la première édition du manuel *Système de comptes de la santé de l'OCDE (2000).*

**Résultats:**

Nous avons retenu 872 rapports sur les CNS provenant de 117 pays. Ces rapports contenaient un total de 2936 matrices pour les quatre types de données. La plupart des pays n'ont pas fourni des données complètes concernant leurs dépenses de santé: seuls 252 des 872 rapports contenaient des données appartenant aux quatre types. Pour la catégorie «non spécifiée par type», trente-huit pays ont indiqué une valeur moyenne supérieure à 20%, quels que soient le type de données et l'année. Plusieurs pays ont fait état de changements conséquents en glissement annuel aussi bien dans le niveau des dépenses de santé que dans leur composition. Ces changements étaient probablement dus à des processus de génération de données. Toutes les données de l'étude sont accessibles au public à l'adresse http://vizhub.healthdata.org/nha/.

**Conclusion:**

Les données provenant des rapports sur les CNS relatives aux dépenses de santé sont souvent incomplètes et, dans certains cas, d'une qualité douteuse. L'amélioration de la qualité des données aiderait les ministères des Finances à allouer des ressources aux systèmes de santé et les ministères de la Santé à mobiliser un capital dans le secteur de la santé tout en permettant aux chercheurs d'établir des comparaisons justes entre les différents systèmes de santé.

## Resumen

**Objetivo:**

Recolectar, compilar y evaluar los informes públicos disponibles sobre las cuentas nacionales de salud (CNS) realizados a nivel mundial entre 1996 y 2010.

**Métodos:**

Se han descargado los informes de las CNS realizados por países de la base de datos de gasto global en salud de la Organización Mundial de la Salud y de la página web StatExtract de la Organización para la Cooperación y el Desarrollo Económicos (OCDE). También se han conseguido informes gracias a Abt Associates, a través de contactos en países específicos y mediante una búsqueda en línea. Se ha recopilado la información siguiendo los cuatro apartados principales que se utilizan en estos informes: (i) fuente de financiamiento; (ii) agente de financiamiento; (iii) función de salud; y (iv) proveedor de salud. Se ha combinado y repasado la información para ajustarse a la primera edición de *Un manual sobre el sistema de cuentas de salud* (2000) de la OCDE.

**Resultados:**

Se han identificado 872 informes de CNS de 117 países, que contienen un total de 2936 matrices para los cuatro apartados. La mayoría de países no tenían información completa sobre el gasto en salud: sólo 252 de los 872 informes contenían información sobre los cuatro apartados. Treinta y ocho países informaron de un valor promedio sin especificar el tipo superior al 20% para la información referente a los cuatro apartados y a todos los años. Algunos países informaron de cambios sustanciales interanuales tanto en el nivel como en la composición del gasto en salud, probablemente debidos a los procesos de generación de datos. Toda la información del estudio está disponible públicamente en http://vizhub.healthdata.org/nha/.

**Conclusión:**

La información de los informes de CNS sobre el gasto en salud es, a menudo, incompleta y, en algunos casos, de calidad cuestionable. Una mejor información ayudaría a los ministerios de finanzas en la asignación de recursos para los sistemas de salud y a los ministerios de sanidad en la asignación de capital para el sector sanitario, y permitiría a los investigadores realizar comparaciones precias entre sistemas de salud.

## ملخص

**الغرض:**

جمع وتصنيف وتقييم تقارير الحسابات الصحية الوطنية المتاحة للعموم (NHA) الصادرة في جميع أنحاء العالم في الفترة من عام **  **1996 وعام **  **2010.

**الطريقة:**

لقد قمنا بتنزيل تقارير الصحة الوطنية الصادرة لكل بلد من قاعدة بيانات نفقات الصحة العالمية في منظمة الصحة العالمية والموقع الإلكتروني StatExtract لمنظمة التعاون الاقتصادي والتنمية (OECD). كما حصلنا أيضًا على تقارير من Abt Associates، من خلال جهات الاتصال القائمة في كل بلد بمفردها ومن خلال البحث مباشرة على شبكة الإنترنت. وقمنا بتصنيف البيانات في الأنواع الأربعة الرئيسية المستخدمة في هذه التقارير: (أ) مصدر التمويل؛ و(ب) وكيل التمويل؛ و(جـ) الوظيفة الصحية؛ و(د) مزود الخدمة الصحية. وقمنا بجمع وتعديل البيانات لتتوافق مع الإصدار الأول من *نظام دليل الحسابات الصحية * الخاص بمنظمة التعاون الاقتصادي والتنمية، (لعام **  **2000).

**النتائج:**

لقد قمنا بتحديد 728 من تقارير الحسابات الصحية الوطنية من 117 **  **بلدًا تحتوي على ما مجموعه 3629 **  **مصفوفة من المصفوفات الخاصة بأنواع البيانات الأربعة. لم توفر معظم البلدان بيانات كاملة لنفقات الصحة: فقد اقتصر عدد التقارير التي تحتوي على بيانات تشمل جميع الأنواع الأربعة على 252 **  **تقريرًا من إجمالي التقارير البالغ عددها 872 تقريرًا. وقد وردت تقارير من ثمانية وثلاثون بلدًا بشأن متوسط قيمة غير محددة حسب النوع يزيد عن 20‏% **  **لجميع أنواع وسنوات البيانات. ووردت تقارير من بعض البلدان بشأن حدوث تغيرات كبيرة سنويًا في مستوى النفقات الصحية وتكوينها والتي يُرجح أن تكون قد صدرت من خلال عمليات لتوليد البيانات. وتتوفر جميع البيانات الخاصة بالدراسة للاطلاع العمومي عليها من خلال الموقع الإلكتروني http://vizhub.healthdata.org/nha/ .

**الاستنتاج:**

عادةً ما تكون تقارير الحسابات الصحية الوطنية عن النفقات الصحية غير مكتملة، بل وتكون جودتها النوعية موضع تساؤل في بعض الحالات. ومن شأن تحسين نوعية البيانات أن تساعد وزارات المالية على تخصيص الموارد اللازمة لنظم الرعاية الصحية، ومساعدة وزارات الصحة في تخصيص رأس المال داخل القطاع الصحي، وتمكين الباحثين من إجراء مطابقات دقيقة بين نظم الرعاية الصحية.

## 摘要

**目的:**

旨在收集、编辑并评估于 1996 年至 2010 年期间在全球制定的公开性国家卫生账户 (NHA) 报告。

**方法:**

我们从世界卫生组织的全球卫生支出数据库以及经济合作与发展组织 (OECD) 的 StatExtract 网站中下载了由国家制定的 NHA 报告。 我们还通过个别国家的联系人和在线搜索获得了来自 Abt Associates 的报告。 我们编辑数据时是按照这些报告中采用的四种主要类型： (i) 融资来源； (ii) 融资机构； (iii) 卫生职能；和 (iv) 卫生提供方。 我们将数据进行合并与调整，使其符合经济合作与发展组织 (OECD) 发布的第一版《卫生核算体系手册》(*A system of health accounts manual) *(2000)。

**结果:**

我们鉴定了来自 117 个国家的 872 份 NHA 报告，其中一共包含四种数据类型的 2936 种矩阵形式。 大部分国家并没有提供完整的卫生支出数据： 在 872 份报告中，只有 252 份包含所有四种类型的数据。 三十八个国家针对所有数据类型和年份报告了一个大于 20%、未指定类别的平均数值。 针对可能通过数据生成流程产生的卫生支出的水平和构成，一些国家报告了大量与上年同期相比的变化。 所有研究数据公布于 http://vizhub.healthdata.org/nha/。

**结论:**

NHA 报告中的卫生支出数据通常是不完整的，并且在一些情况下，质量也有问题。 如果数据更好，将不仅有助于财务部向卫生系统分配资源，并有助于卫生部在卫生部门内分配资本，而且能使研究人员在卫生系统之间进行更加精准的对比。

## Резюме

**Цель:**

Собрать, скомпилировать и оценить публично доступные национальные счета сектора здравоохранения (НССЗ), созданные во всем мире в период с 1996 по 2010 год.

**Методы:**

Мы получили сгенерированные по каждой из стран отчеты о НССЗ из мировой базы расходов на здравоохранение Всемирной организации здравоохранения и с веб-сайта Организации по экономическому сотрудничеству и развитию (ОЭСР) StatExtract. Мы также получили отчеты от компании Abt Associates путем личных контактов в отдельных странах и посредством поиска в сети. Мы скомпилировали данные по четырем основным типам, использованным в данных отчетах: (i) источник финансирования; (ii) кредитующая организация; (iii) подразделение здравоохранения и (iv) организация, оказывающая услуги здравоохранения. Мы скомбинировали и откорректировали данные так, чтобы соответствовать рекомендациям, изложенным в первом издании *Справочника по системам национальных счетов в секторе здравоохранения* ОЭСР (2000 год).

**Результаты:**

Нами были выявлены 872 отчета НССЗ из 117 стран, в которых содержалось всего 2936 таблица для четырех типов данных. Большинство стран не предоставляют полных данных по затратам на здравоохранение: данные всех четырех типов содержались только в 252 из 872 отчетов. Тридцать восемь стран сообщили об уровне расходов, значение которого (без указания характера) в среднем составляет свыше 20% для всех типов данных в каждом году. Некоторые страны сообщали о значительных изменениях, происходивших год от года как в уровне, так и в составе затрат на здравоохранение, которые, вероятно, были результатом процессов генерирования данных. Все данные исследования доступны для общественности по ссылке http://vizhub.healthdata.org/nha/.

**Вывод:**

Данные из отчетов НССЗ по затратам на здравоохранение часто неполны и в некоторых случаях имеют сомнительное качество. Улучшение качества данных позволило бы министерствам финансов выделять ресурсы системам здравоохранения, помогать министерствам здравоохранения в выделении средств в секторе охраны здоровья и дало бы возможность исследователям проводить точные сравнения разных систем здравоохранения между собой.

## Introduction

Expenditure on health makes up a substantial part of the global economy. The share of resources allocated worldwide to health is increasing faster than ever^1^ and health expenditure per person is rising in almost every country. Both government expenditure on – and development assistance for – health have continued to grow despite the global financial crisis.^2^^–^^5^ Although trends in total health expenditure are reasonably well documented, less is known about how that money is actually spent. To control costs and maintain the effectiveness of the health system, policy-makers, health administrators and medical professionals need detailed information on financing sources, health-care delivery and health-care providers. In particular, precise and disaggregated health expenditure data are required.

Attempts to quantify national health expenditure began as early as 1926 in the United States of America, led by the American Medical Association.^6^ In the 1970s, the Organisation for Economic Co-operation and Development (OECD) led an international effort to collect consistent health expenditure data and, in the 1980s, OECD launched DataWatch, which collected data on health-care expenditure, use and outcomes from 24 OECD countries.^7^ To standardize data on health expenditure and resource flows, the OECD published *A system of health accounts, first edition* (SHA 2000)^8^ in 2000 and introduced the International Classification of Health Accounts. Building on SHA 2000, the OECD worked with the World Health Organization (WHO) and Eurostat to publish *A system of health accounts, 2011 edition* (SHA 2011).^9^ The SHA framework is the most widely-used reference for health expenditure accounting. The SHA manuals give instructions on how to categorize a country’s expenditure as health expenditure for a given year by defining health activities, setting time intervals and establishing residency definitions.^10^ Suggested data sources include budgets, censuses, surveys, tax reports, trade statistics, government documents and reports from nongovernmental organizations.

OECD and individual countries have applied the SHA framework to produce information on national health expenditure in a form collectively known as national health accounts (NHAs). These NHAs aim to provide systematic, comprehensive and consistent data on health system resource flows and can, therefore, serve as canonical sources of disaggregated information on health expenditure.^10^^–^^13^ In line with the *Guide to producing national health accounts* released in 2003,^14^ NHAs classify national expenditure on health by addressing four questions: (i) Where do health resources come from? (i.e. What is the financing source?); (ii) Who manages spending? (i.e. Who is the financing agent?); (iii) What goods and services are purchased? (i.e. What is the health function?); and (iv) Who provides which services? (i.e. Who is the health provider?). The NHAs are designed to answer these questions within a standardized framework and NHA reports are regarded as the international standard for tracking health resources.^9^^,^^12^^,^^15^ Consequently, NHAs can be useful for cross-country analyses.

However, no complete international set of data associated with these four questions has been available to date. Forty-four countries have routinely produced NHA reports since 2010^11^ – most are OECD member states. The OECD requests member states to submit NHAs each year using the SHA framework but there is no legal obligation for countries to comply. Since 2003, most NHA data on OECD countries have come from a questionnaire produced jointly by the OECD, Eurostat and WHO –a survey completed in each country and validated to ensure data comparability between countries.^16^^,^^17^ OECD, the World Bank and WHO have held joint meetings to encourage developing countries to institutionalize the production of NHAs. However, few countries systematically produce NHAs and often their health reports do not follow the SHA framework, which reduces data comparability. Nevertheless, WHO has collected data sets from 14 countries and 41 data sets are available as NHA tables in the WHO global health expenditure database,^18^ but the collection is not complete. This database also provides information on financing sources and financing agents under NHA indicators. Although an attempt has been made to build a comprehensive database using adjustments, estimates and projections to correct misreported data, fill in missing data and cover more recent years,^13^ a complete series covering all four categories of data is still lacking. Furthermore, it is unclear which data and methods were used to impute data to fill in the many gaps in country-generated NHAs.

Our aims were to supplement WHO’s database by compiling and reviewing all NHA reports produced worldwide between 1996 and 2010 and to make information on the four financial data types publicly available for all countries through our online visualization tool (available at: http://vizhub.healthdata.org/nha/). The tool is intended to provide a centralized source of country-generated data that can be consulted without the need for statistical inference.

## Methods

We carried out a search for reports on country-generated NHAs covering the period 1996 to 2010. Eligible reports contained information at the national level on expenditure across the whole health-care system and used the SHA 2000 or the health satellite accounts of the system of national accounts framework.^19^ The system national accounts framework is similar to the NHA framework except that it is managed by a country’s national income accounts office and encompasses all sectors of the economy. Satellite accounts are used when there is interest to further disaggregate a specific sector. We excluded subnational data, accounts of specific diseases and reports produced exclusively by third parties. Data were collected from a variety of sources that were available between 1 January 2010 and 14 April 2014 – a complete list of the NHAs reviewed is available from the corresponding author on request. We downloaded all NHAs available on the WHO global health expenditure database^18^ and the OECD StatExtracts website,^20^ which reports health and financing expenditure data for the 34 OECD member countries. We also received 51 NHAs directly from Abt Associates – a company that provides technical assistance to countries in producing NHAs. Additionally, NHAs were obtained directly from contacts in individual countries and reports were identified through an online search on Google using the terms “national health accounts,” “NHAs”, “system of health accounts”, “SHA 2000”, “SHA 1.0”, “SHA 2011”, “SHA 2.0”, “system of national accounts”, “SNAs” and “satellite health accounts”. Data were downloaded from the websites of ministries of health, USAID and other governmental and development organizations and were reported in accordance with the preferred reporting items for systematic reviews and meta-analyses.^21^

We extracted data from four NHA types: (i) financing agent by financing source; (ii) health function by financing agent; (iii) health function by health provider; and (iv) health provider by financing agent. We also extracted and analysed data on any of these NHA types that were available in an NHA but not presented in matrix form. In addition, we included country data presented on part of any one of the matrices even if that matrix was incomplete. We formatted the matrices and comparable data extracted from the NHA reports in accordance with the SHA 2000 framework. However, because NHA reports were produced by individual countries, there was a wide variation in the subcategories used both between countries and in different years. We took this heterogeneity into account and fitted the reported data to the framework by creating a template that included all subcategories used in at least one data source. More information on these subcategories and their classification within individual NHAs is available from the corresponding author on request.

We collected data in the most disaggregated form available. Then, we aggregated the data into the broadest categories within each of the four types. We retained the value of the total health expenditure reported and, when the sum of the values of the aggregated data did not equal the reported total, we accounted for the difference by assigning values to not-specified-by-kind components. Our final data set included two not-specified-by-kind categories: given and generated. The given category was the component of a country’s reported health expenditure that did not clearly fall within one of the SHA categories; the generated category was the component we created when the sum of values for individual categories within a type did not equal the reported total for that type. A positive value for the generated not-specified-by-kind component indicated that the sum of the categories was less than the reported total (i.e. the country underreported data at the item level) and a negative value indicated that the sum was greater than the reported total (i.e. the country overreported data at the item level). If there was more than one source of data for a given type for a country and year (e.g. for the financing agent, both the financing-agent-by-financing-source matrix and the health-provider-by-financing-agent matrix may have been available), only data from one source were included in the final data set. We ranked each of the matrices, or one-dimensional tables if the full matrices were not given, for each type according to their completeness and the perceived reliability of the data. Then, these rankings were used to select the most reliable data for compiling the data set. To enable between-country comparisons, all expenditure was converted into United States dollars (US$) at 2010 values (more information on the compilation and exchange methods is available from the corresponding author).

We examined trends in NHA reporting across all countries between 1996 and 2010 for each of the four types and we determined the number of missing type matrices for each country. The quality of the data provided was evaluated by examining the size of both the given and generated not-specified-by-kind components for each country and year. Finally, we analysed trends in components of national health expenditure across countries and time for each type in terms of both the absolute level of expenditure and the percentage of total health expenditure.

## Results

Our search identified 872 NHA reports from 117 countries containing a total of 2936 matrices or tables. Some economic indicators, such as gross domestic product, were available for almost all countries and years and included component breakdowns.^22^ Although data on total health expenditure and health expenditure per capita were available for most countries, details of the components of this expenditure were not available because many countries did not produce NHA reports. However, of the 872 NHA reports we identified, only 252 presented data using all four types of matrices for any country and year. Table 1 (available at: http://www.who.int/bulletin/volumes/93/14/07-145235) provides a summary of the reports available for each country and year. Our data can also be accessed at http://vizhub.healthdata.org/nha/ using an interactive visualization tool.

**Table 1:** National health accounts,^a^ by country and year, 1996–2010

Country	No. of national health accounts reports produced															
	1996	1997	1998	1999	2000	2001	2002	2003	2004	2005	2006	2007	2008	2009	2010	Total
Afghanistan	0	0	0	0	0	0	0	0	0	0	0	0	1	0	0	1
Albania	0	0	0	0	0	0	0	1	0	0	0	1	1	1	0	4
Argentina	1	1	1	1	0	0	0	0	0	0	0	0	0	0	0	4
Armenia	0	0	0	0	0	0	0	0	1	1	1	1	0	0	0	4
Australia^b^	1	1	1	1	1	1	1	1	1	1	1	1	1	1	1	15
Austria^b^	1	1	1	1	1	1	1	1	1	1	1	1	1	1	1	15
Bangladesh	1	1	1	1	1	1	1	1	1	1	1	1	0	0	0	12
Belgium^b^	1	1	1	1	1	1	1	1	1	1	1	1	1	1	1	15
Benin	0	0	0	0	0	0	0	1	0	0	0	0	0	0	0	1
Bhutan	0	0	0	0	0	0	0	0	0	0	0	0	0	1	0	1
Bolivia (Plurinational State of)	1	0	0	0	0	0	0	0	0	0	0	0	0	0	0	1
Botswana	0	0	0	0	0	0	0	0	0	0	0	1	1	1	0	3
Brazil	0	0	0	0	1	1	1	1	1	1	1	1	1	1	0	10
Bulgaria	0	0	0	0	0	0	0	1	1	1	1	1	0	0	0	5
Burkina Faso	0	0	0	0	0	0	0	1	1	1	1	1	1	1	0	7
Burundi	0	0	0	0	0	0	0	0	0	0	0	1	0	0	0	1
Cabo Verde	0	0	0	0	0	0	0	0	0	0	0	0	1	1	0	2
Canada^b^	1	1	1	1	1	1	1	1	1	1	1	1	1	1	1	15
Chile^b^	1	1	1	1	1	1	1	1	1	1	1	1	1	1	1	15
China	1	1	1	1	1	1	1	1	1	1	1	1	1	1	1	15
Colombia	0	0	0	0	1	1	1	1	0	0	0	0	0	0	0	4
Côte d'Ivoire	0	0	0	0	0	0	0	0	0	0	0	1	1	0	0	2
Czech Republic^b^	1	1	1	1	1	1	1	1	1	1	1	1	1	1	1	15
Democratic Republic of the Congo	0	0	0	0	0	0	0	0	0	0	0	0	1	1	1	3
Denmark^b^	1	1	1	1	1	1	1	1	1	1	1	1	1	1	1	15
Dominican Republic	1	1	1	1	1	1	1	1	1	1	1	1	1	0	0	13
Ecuador	0	0	0	0	0	0	0	0	1	1	0	0	0	0	0	2
Egypt	0	0	0	0	0	1	0	0	0	0	0	1	1	0	0	3
El Salvador	0	0	0	1	1	0	0	0	0	0	0	0	0	0	0	2
Estonia^b^	0	0	0	1	1	1	1	1	1	1	1	1	1	1	1	12
Ethiopia	0	0	0	1	0	0	0	0	1	0	0	1	0	0	0	3
Fiji	0	0	0	0	0	0	0	0	0	0	0	1	1	1	1	4
Finland^b^	1	1	1	1	1	1	1	1	1	1	1	1	1	1	1	15
France^b^	1	1	1	1	1	1	1	1	1	1	1	1	1	1	1	15
Gambia	0	0	0	0	0	0	1	1	1	0	0	0	0	0	0	3
Georgia	0	0	0	0	0	1	1	1	1	1	1	1	1	1	0	9
Germany^b^	1	1	1	1	1	1	1	1	1	1	1	1	1	1	1	15
Ghana	0	0	0	0	0	0	1	0	0	0	0	0	0	0	0	1
Greece^b^	1	1	1	1	1	1	1	1	1	1	1	1	1	1	1	15
Guatemala	1	1	1	0	0	0	0	0	0	0	0	0	0	0	0	3
Honduras	0	0	1	0	0	0	0	0	0	0	0	0	0	0	0	1
Hungary^b^	1	1	1	1	1	1	1	1	1	1	1	1	1	1	1	15
Iceland^b^	1	1	1	1	1	1	1	1	1	1	1	1	1	1	1	15
India	0	0	0	0	0	1	0	0	1	0	0	0	0	0	0	2
Indonesia	0	0	0	0	0	0	0	0	0	1	1	1	1	1	0	5
Iran (Islamic State of)	0	1	1	0	0	1	1	1	1	1	1	1	1	0	0	10
Ireland^b^	1	1	1	1	1	1	1	1	1	1	1	1	1	1	1	15
Israel^b^	1	1	1	1	1	1	1	1	1	1	1	1	1	1	1	15
Italy^b^	1	1	1	1	1	1	1	1	1	1	1	1	1	1	1	15
Japan^b^	1	1	1	1	1	1	1	1	1	1	1	1	1	1	1	15
Jordan	0	0	1	0	1	1	0	0	0	0	0	1	1	1	0	6
Kenya	0	0	0	0	0	1	0	0	0	1	0	0	0	1	0	3
Kiribati	0	0	0	0	0	0	0	0	0	0	0	1	1	1	0	3
Kyrgyzstan	0	0	0	0	0	0	0	0	1	0	1	1	1	1	0	5
Lao People's Democratic Republic	0	0	0	0	0	0	0	0	0	0	0	0	0	1	0	1
Lebanon	0	0	1	0	0	0	0	0	0	0	0	0	0	0	0	1
Liberia	0	0	0	0	0	0	0	0	0	0	0	1	0	1	0	2
Luxembourg^b^	1	1	1	1	1	1	1	1	1	1	1	1	1	1	1	15
Madagascar	0	0	0	0	0	0	0	1	0	0	0	1	0	0	0	2
Malawi	0	0	1	0	0	0	1	0	0	1	1	1	1	0	0	6
Malaysia	0	1	1	1	1	1	1	1	1	1	1	1	1	1	0	13
Mali	0	0	0	1	1	1	1	1	1	0	0	0	0	0	0	6
Mexico^b^	1	1	1	1	1	1	1	1	1	1	1	1	1	1	1	15
Micronesia (Federated States of)	0	0	0	0	0	0	0	0	0	1	1	1	1	0	0	4
Mongolia	0	0	0	0	0	0	1	0	0	0	0	0	0	0	0	1
Montenegro	0	0	0	0	0	0	0	0	1	1	1	0	0	0	0	3
Morocco	0	1	0	0	0	1	0	0	0	0	1	0	0	0	1	4
Mozambique	0	0	0	0	0	0	0	0	1	1	1	0	0	0	0	3
Myanmar	0	0	0	0	0	0	1	1	1	1	1	1	0	0	0	6
Namibia	0	0	0	1	1	1	1	1	1	1	1	1	1	0	0	10
Nepal	0	0	0	0	1	1	1	1	1	1	1	1	1	0	0	9
Netherlands^b^	1	1	1	1	1	1	1	1	1	1	1	1	1	1	1	15
New Zealand^b^	1	1	1	1	1	1	1	1	1	1	1	1	1	1	1	15
Nicaragua	1	1	1	1	1	1	1	0	0	0	0	0	0	0	0	7
Niger	0	0	0	0	0	0	0	1	0	0	0	0	0	0	0	1
Nigeria	0	0	1	1	1	1	1	1	1	1	0	0	0	0	0	8
Norway^b^	1	1	1	1	1	1	1	1	1	1	1	1	1	1	1	15
Oman	0	0	1	0	0	0	0	0	0	0	0	0	0	0	0	1
Pakistan	0	0	0	0	0	0	0	0	0	1	0	1	0	0	0	2
Palau	0	0	0	0	0	0	0	0	0	0	0	1	0	0	0	1
Papua New Guinea	0	0	1	1	1	0	0	0	0	0	0	0	0	0	0	3
Paraguay	0	1	1	1	1	1	0	0	0	0	0	0	0	0	0	5
Peru	1	0	0	0	0	0	0	0	0	0	0	0	0	0	0	1
Philippines	1	1	1	1	1	1	1	1	1	1	1	1	1	1	1	15
Poland^b^	1	1	1	1	1	1	1	1	1	1	1	1	1	1	1	15
Portugal^b^	1	1	1	1	1	1	1	1	1	1	1	1	1	1	1	15
Qatar	0	0	0	0	0	0	0	0	0	0	0	0	0	1	1	2
Republic of Korea^b^	1	1	1	1	1	1	1	1	1	1	1	1	1	1	1	15
Rwanda	0	0	1	0	1	0	1	1	0	0	1	0	0	0	0	5
Samoa	0	0	0	0	0	0	1	0	1	0	1	0	0	0	0	3
Senegal	0	0	0	0	0	0	0	0	0	1	0	0	0	0	0	1
Serbia	0	0	0	0	0	0	0	1	1	1	1	1	1	0	0	6
Seychelles	0	0	0	0	0	0	0	0	0	0	0	0	0	1	0	1
Sierra Leone	0	0	0	0	0	0	0	0	1	1	1	1	1	1	1	7
Slovakia^b^	0	1	1	1	1	1	1	1	1	1	1	1	1	1	1	14
Slovenia^b^	1	1	1	1	1	1	1	1	1	1	1	1	1	1	1	15
South Africa	1	1	1	0	0	0	0	0	0	0	0	0	0	0	0	3
Spain^b^	1	1	1	1	1	1	1	1	1	1	1	1	1	1	1	15
Sri Lanka	1	1	1	1	1	1	1	1	1	1	1	0	0	0	0	11
Suriname	0	0	0	0	1	0	0	0	0	0	1	0	0	0	0	2
Sweden^b^	1	1	1	1	1	1	1	1	1	1	1	1	1	1	1	15
Switzerland^b^	1	1	1	1	1	1	1	1	1	1	1	1	1	1	1	15
Thailand	1	1	1	1	1	1	1	1	1	1	1	1	1	1	1	15
Togo	0	0	0	0	0	0	0	0	0	0	0	0	1	0	0	1
Tonga	0	0	0	0	0	1	0	1	0	1	0	0	0	0	0	3
Tunisia	0	0	0	0	0	0	0	0	1	1	0	0	0	0	0	2
Turkey^b^	1	1	1	1	1	1	1	1	1	1	1	1	1	0	0	13
Uganda	0	1	1	1	1	0	0	0	0	0	0	0	0	0	0	4
Ukraine	0	0	0	0	0	0	0	1	1	0	0	0	0	0	0	2
United Kingdom^b^	1	1	1	1	1	1	1	1	1	1	1	1	1	1	1	15
United Republic of Tanzania	0	0	0	1	0	0	0	0	0	0	0	0	0	1	0	2
United States (the)^b^	1	1	1	1	1	1	1	1	1	1	1	1	1	1	1	15
Uruguay	0	1	1	0	0	0	0	0	1	1	1	1	1	0	0	7
Vanuatu	0	0	0	0	0	0	0	0	0	1	0	1	0	0	0	2
Yemen	0	0	1	0	0	0	0	1	0	0	1	1	0	0	0	4
Zambia	1	1	1	1	1	1	1	1	1	1	1	0	0	0	0	11
Zimbabwe	0	0	0	1	0	0	0	0	0	0	0	0	0	0	0	1
**Total**	**45**	**50**	**58**	**54**	**56**	**58**	**58**	**63**	**66**	**67**	**65**	**71**	**63**	**57**	**41**	**872**

^a^ Excludes satellite health accounts and national health accounts subaccounts.

^b^ Member of the Organisation for Economic Co-operation and Development.

**Fig. 1:**
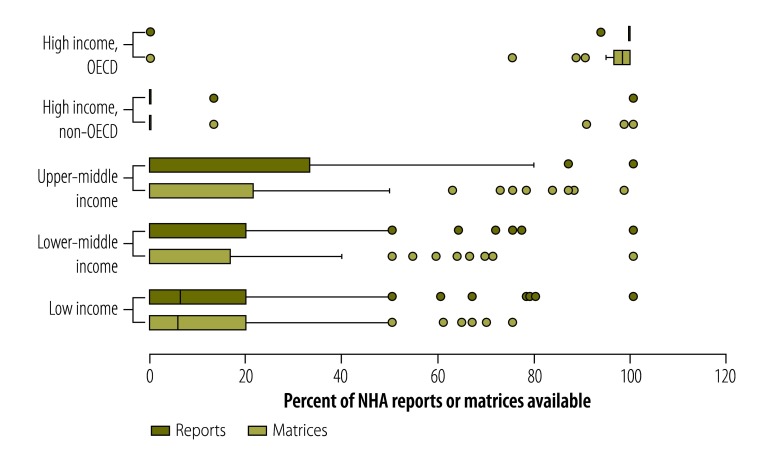
National health accounts reports and data type matrices available, by country income group,^a^ 1996–2010

NHA: national health accounts; OECD: Organisation for Economic Co-operation and Development.

^a^ Country income categories were defined using historical World Bank income classifications.^23^

Notes: The maximum number of reports that could have been available for each country was 15: one for each year from 1996 to 2010. The maximum number of matrices or tables for the four main data types (i.e. financing agent, financing source, health function and health provider) that could have been available for each country was 60: four for each of 15 years. In the box plot, the box indicates the first and third quartiles, the whiskers extend to 1.5 times the interquartile range, the vertical line is the median and the points represent outliers.

**Fig. 2:**
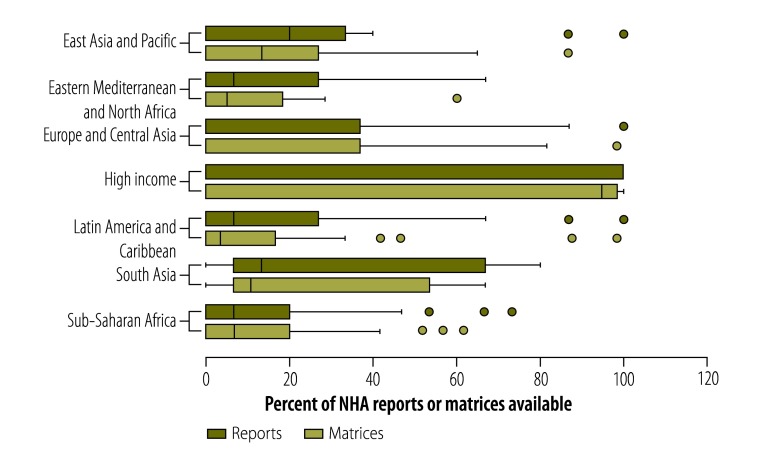
National health accounts reports and data type matrices available, by geographical areas,^a^ 1996–2010

Fig. 1 shows the number of NHA reports and type matrices produced by countries in different income categories (as defined by the World Bank^23^ and assigned historically for each country and year) as a percentage of the maximum possible: the maximum number of reports that could have been available for each country was 15 (i.e. one for each year from 1996 to 2010) and the maximum number of type matrices was 60 (i.e. four in each of 15 years). High-income countries belonging to the OECD produced most NHAs: the median proportion of reports available for these countries was 100% and the median proportion of matrices available was 98%. In contrast, non-OECD, high-income countries typically produced no reports and only a few matrices. The interquartile ranges in the box plots in Fig. 1 show that the number of reports available was lowest for lower-middleincome and low-income countries. Despite having fewer resources, low-income countries produced a comparable number (average of 1.88 reports per country, across all years) of reports to middle-income countries (average of 1.86 reports per country, across all years). For upper-middle income and lower-middle income countries, the median number of tables reported over the 15-year period was 0. Out of the 193 United Nations’ Members States, 76 Member States did not produce any reports; 57% of these countries were classified as upper-middle income or lower-middle income. Fig. 2 shows the number of NHA reports and type matrices produced by countries in different geographical areas: the numbers were lowest for countries in areas of the Eastern Mediterranean and North Africa, Latin America and the Caribbean, and sub-Saharan Africa.

NHA: national health accounts.

^a^ The countries were grouped by their income or regional classification as designated by the World Bank in the year of reporting.^23^ The countries included in the geographical groupings were all low- or middle-income countries. Countries remained in the same grouping from 1996 to 2010 unless otherwise stated. East Asia and Pacific included Cambodia, China, Democratic People's Republic of Korea, Fiji, Indonesia, Kiribati, Lao People's Democratic Republic, Malaysia, Marshall Islands, Micronesia (Federated States of), Mongolia, Myanmar, Palau, Papua New Guinea, Philippines, Samoa, Solomon Islands, Thailand, Timor-Leste, Tonga, Vanuatu and Viet Nam. Eastern Mediterranean and North Africa included Algeria, Bahrain (1996–2000), Djibouti, Egypt, Iran (Islamic State of), Iraq, Jordan, Lebanon, Libya, Morocco, Oman (1996–2006), Saudi Arabia (1996–2003), Syrian Arab Republic, Tunisia and Yemen. Europe and Central Asia included Albania, Armenia, Azerbaijan, Belarus, Bosnia and Herzegovina, Bulgaria, Croatia (1996–2007), Estonia (1996–2005), Georgia, Hungary, Kazakhstan, Kyrgyzstan, Latvia (1996–2008, 2010), Lithuania, Malta (1996–1997, 1999, 2001), Montenegro, Republic of Moldova, Romania, Russian Federation, Serbia, Slovakia (1996–1999), Slovenia (1996), Tajikistan, the former Yugoslav Republic of Macedonia, Turkey, Turkmenistan, Ukraine and Uzbekistan. High income included Andorra, Antigua and Barbuda (2002, 2005–2008), Australia, Austria, Bahamas, Bahrain (2001–2010), Barbados (2000, 2002, 2006–2010), Belgium, Brunei Darussalam, Canada, Chile (2010), Croatia (2008–2010), Cyprus, Czech Republic, Denmark, Equatorial Guinea (2007–2010), Estonia (2006–2010), Finland, France, Germany, Greece, Iceland, Ireland, Israel, Italy, Japan, Kuwait, Latvia (2009), Luxembourg, Malta (1998, 2000, 2002–2010), Netherlands, New Zealand, Norway, Oman (2007–2010), Poland, Portugal, Qatar, Republic of Korea, Saudi Arabia (2004–2010), Singapore, Slovakia (2000–2010), Slovenia (1997–2010), Spain, Sweden, Switzerland, Trinidad and Tobago (2006–2010), United Arab Emirates, United Kingdom and the United States. Latin America and Caribbean included Antigua and Barbuda (1996–2001, 2003–2004, 2009–2010), Argentina, Barbados (1996–1999, 2001, 2003–2005), Belize, Bolivia (Plurinational State of), Brazil, Chile (1996–2009), Colombia, Costa Rica, Cuba, Dominica, Dominican Republic, Ecuador, El Salvador, Grenada, Guatemala, Guyana, Haiti, Honduras, Jamaica, Mexico, Nicaragua, Panama, Paraguay, Peru, Saint Lucia, Saint Vincent and the Grenadines, Suriname, Trinidad and Tobago (1996–2005), Uruguay and Venezuela. South Asia included Afghanistan, Bangladesh, Bhutan, India, Maldives, Nepal, Pakistan and Sri Lanka. Sub-Saharan Africa included Angola, Benin, Botswana, Burkina Faso, Burundi, Cabo Verde, Cameroon, Central African Republic, Chad, Comoros, Congo, Côte d'Ivoire, Democratic Republic of the Congo, Equatorial Guinea (1996–2006), Eritrea, Ethiopia, Gabon, Gambia, Ghana, Guinea, Guinea-Bissau, Kenya, Lesotho, Liberia, Madagascar, Malawi, Mali, Mauritania, Mauritius, Mozambique, Namibia, Niger, Nigeria, Rwanda, Sao Tome and Principe, Senegal, Seychelles, Sierra Leone, Somalia, South Africa, South Sudan, Sudan, Swaziland, Togo, Uganda, United Republic of Tanzania, Zambia and Zimbabwe.

Notes: The maximum number of reports that could have been available for each country was 15: one for each year from 1996 to 2010.The maximum number of matrices or tables for the four main data typologies (i.e. financing agent, financing source, health function and health provider) that could have been available for each country was 60: four for each of 15 years. In the box plot, the box indicates the first and third quartiles, the whiskers extend to 1.5 times the interquartile range, the vertical line is the median and the points represent outliers.

Table 2 shows how the number of NHA reports produced by countries in different income categories varied over time. Despite efforts to institutionalize NHA production in 2000 and 2003, only 20% (434/2193) of country-year NHA reports from upper-middle income, lower-middle income and low-income countries were produced between 1996 and 2010. Moreover, only 20% (4/20) of non-OECD, high-income countries produced a report in their most productive year, 2009. Reporting lag probably explains the low number of reports available for 2010.

**Table 2:** Countries producing national health accounts, by income group, 1996–2010

Historical country income group^a^	% of countries producing national health accounts reports														
	1996	1997	1998	1999	2000	2001	2002	2003	2004	2005	2006	2007	2008	2009	2010
OECD, high-income	100	100	100	100	100	100	100	100	100	100	100	100	96	100	97
Non-OECD, high-income	11	20	18	20	17	18	14	17	15	14	18	16	15	20	6
Upper-middle income	24	30	24	23	23	19	18	20	26	26	29	32	28	20	8
Lower-middle income	11	16	18	19	23	28	21	28	29	30	26	28	25	17	6
Low-income	7	6	16	14	13	13	20	21	22	22	20	28	18	20	6

OECD: Organisation for Economic Co-operation and Development.

^a^ Country income categories were defined using historical World Bank income classifications.^23^

### Unspecified data

**Fig. 3:**
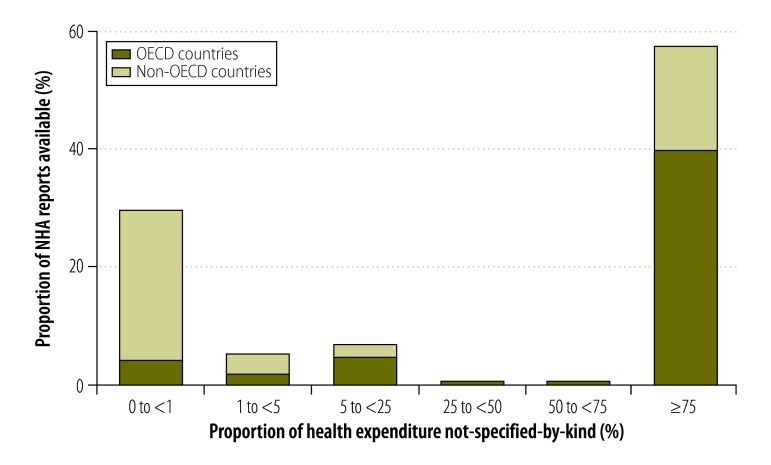
Proportion of a country’s health expenditure not-specified-by-kind^a^ in national health accounts financing source matrices, 1996–2010

NHA: national health accounts; OECD: Organisation for Economic Co-operation and Development.

^a^ Health expenditure was categorized as not-specified-by-kind if: (i) the expenditure was listed in a country-defined expenditure category that did not clearly fall within one of the expenditure categories defined by *A system of health accounts*^8^ (i.e. given not-specified-by-kind); or (ii) the sum of the values for individual categories within a financing source matrix did not equal the reported total for all financing sources – in this case, the not-specified-by-kind expenditure was the difference between the reported total and the sum (i.e. generated not-specified-by-kind).

**Fig. 4:**
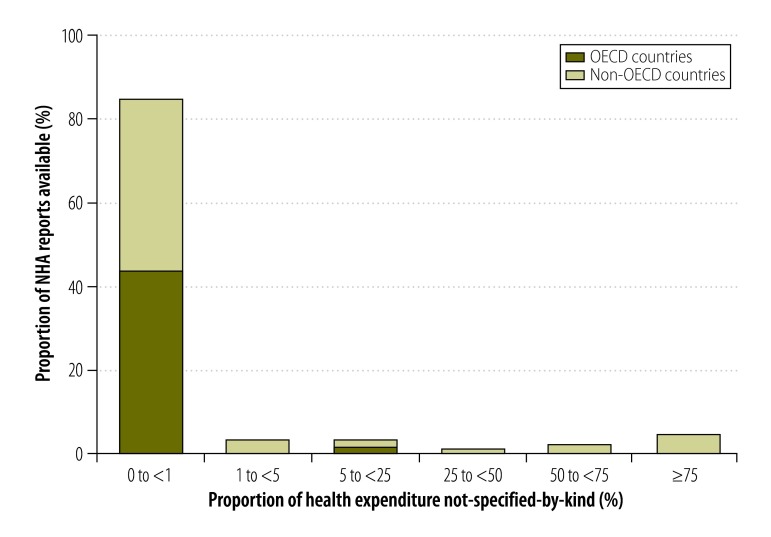
Proportion of a country’s health expenditure not-specified-by-kind^a^ in national health accounts financing agent matrices, 1996–2010

NHA: national health accounts; OECD: Organisation for Economic Co-operation and Development.

^a^ Health expenditure was categorized as not-specified-by-kind if: (i) the expenditure was listed in a country-defined expenditure category that did not clearly fall within one of the expenditure categories defined by *A system of health accounts*^8^ (i.e. given not-specified-by-kind); or (ii) the sum of the values for individual categories within a financing agent matrix did not equal the reported total for all financing agents – in this case, the not-specified-by-kind expenditure was the difference between the reported total and the sum (i.e. generated not-specified-by-kind).

**Fig. 5:**
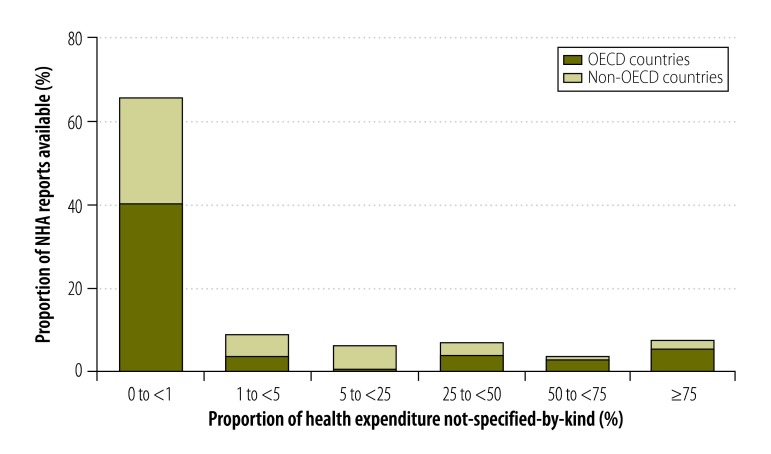
Proportion of a country’s health expenditure not-specified-by-kind^a^ in national health accounts health function matrices, 1996–2010

NHA: national health accounts; OECD: Organisation for Economic Co-operation and Development.

^a^ Health expenditure was categorized as not-specified-by-kind if: (i) the expenditure was listed in a country-defined expenditure category that did not clearly fall within one of the expenditure categories defined by *A system of health accounts*^8^ (i.e. given not-specified-by-kind); or (ii) the sum of the values for individual categories within a health function matrix did not equal the reported total for all health functions – in this case, the not-specified-by-kind expenditure was the difference between the reported total and the sum (i.e. generated not-specified-by-kind).

**Fig. 6:**
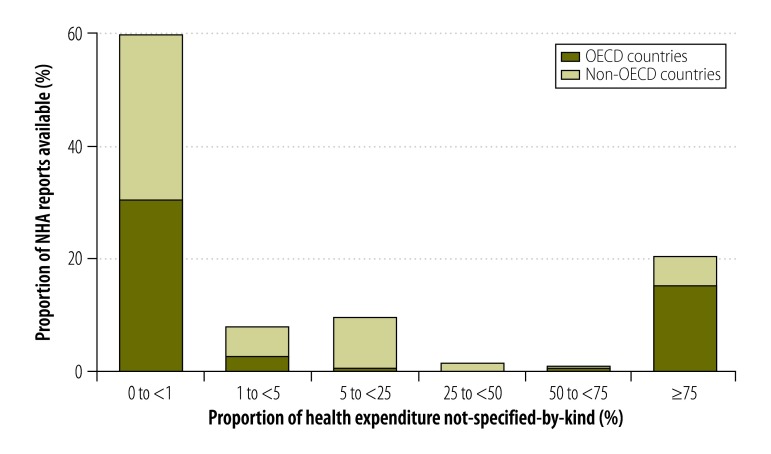
Proportion of a country’s health expenditure not-specified-by-kind^a^ in national health accounts health provider matrices, 1996–2010

NHA: national health accounts; OECD: Organisation for Economic Co-operation and Development.

^a^ Health expenditure was categorized as not-specified-by-kind if: (i) the expenditure was listed in a country-defined expenditure category that did not clearly fall within one of the expenditure categories defined by *A system of health accounts*^8^ (i.e. given not-specified-by-kind); or (ii) the sum of the values for individual categories within a health provider matrix did not equal the reported total for all health providers – in this case, the not-specified-by-kind expenditure was the difference between the reported total and the sum (i.e. generated not-specified-by-kind).

**Fig. 7:**
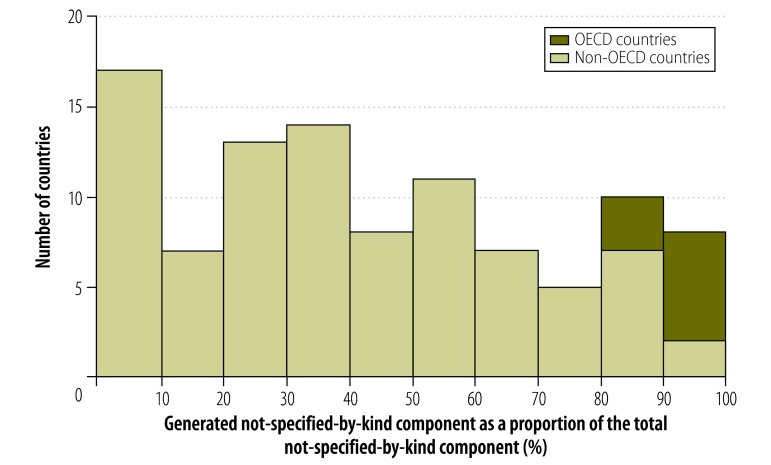
Generated not-specified-by-kind component^a^ of health expenditure as a share of the total not-specified-by-kind component,^b^ 1996–2010

In some countries, the combination of the given and generated not-specified-by-kind components made up more than 75% of expenditure reported in financing source matrices (Fig. 3). The proportions for other data types are shown in Fig. 4, Fig. 5 and Fig. 6 (available at: http://www.who.int/bulletin/volumes/93/14/07-145235). In deriving these figures, we used the total number of NHAs available in the denominator, even if the breakdowns of type matrices were not provided. For example, China reported totals for its health provider matrices but gave no details of components. Consequently, 100% of expenditure reported by health provider type was categorized as not-specified-by-kind. Overall, the size of the generated not-specified-by-kind component was greatest for financing source matrices and health provider matrices. Data on financing sources were produced infrequently. Fig. 7 shows the magnitude of the generated not-specified-by-kind component as a percentage of the total not-specified-by-kind component. For each country, the percentage was estimated across all available matrices. The generated not-specified-by-kind component was at least 50% of the total not-specified-by-kind component for 46% (54/117) of countries. For all OECD countries for which a value for the generated not-specified-by-kind component was required, that component comprised more than 80% of the total not-specified-by-kind component. For OECD countries, therefore, the not-specified-by-kind component was mostly needed to compensate for the sum of components within a matrix not equalling the total.

OECD: Organisation for Economic Co-operation and Development.

^a^ The generated not-specified-by-kind component of health expenditure was the value we generated when the sum of the reported values of individual categories within a data type (i.e. financing agent, financing source, health function or health provider) did not equal the total for that type.

^b^ The total not-specified-by-kind component was the sum of the generated and given not-specified-by-kind components, where the given not-specified-by-kind component was the component of a country’s total health expenditure that did not clearly fall within one of the categories specified by the publication *A system of health accounts*.^8^

### Year-on-year changes

**Fig. 8:**
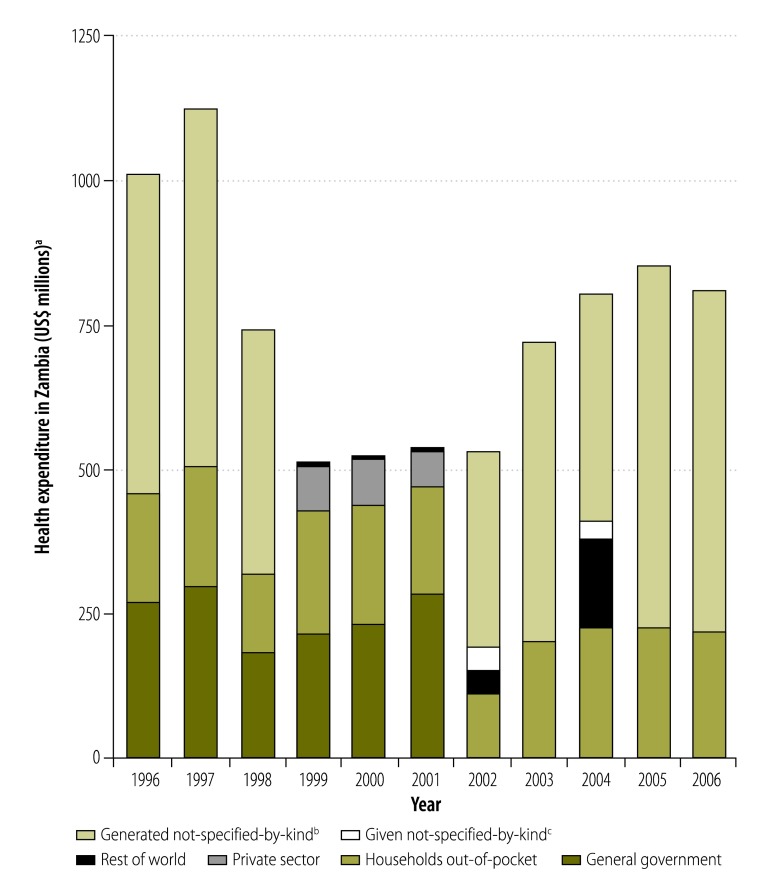
Health expenditure in Zambia, categorized by financing agent, 1996–2006

**Fig. 9:**
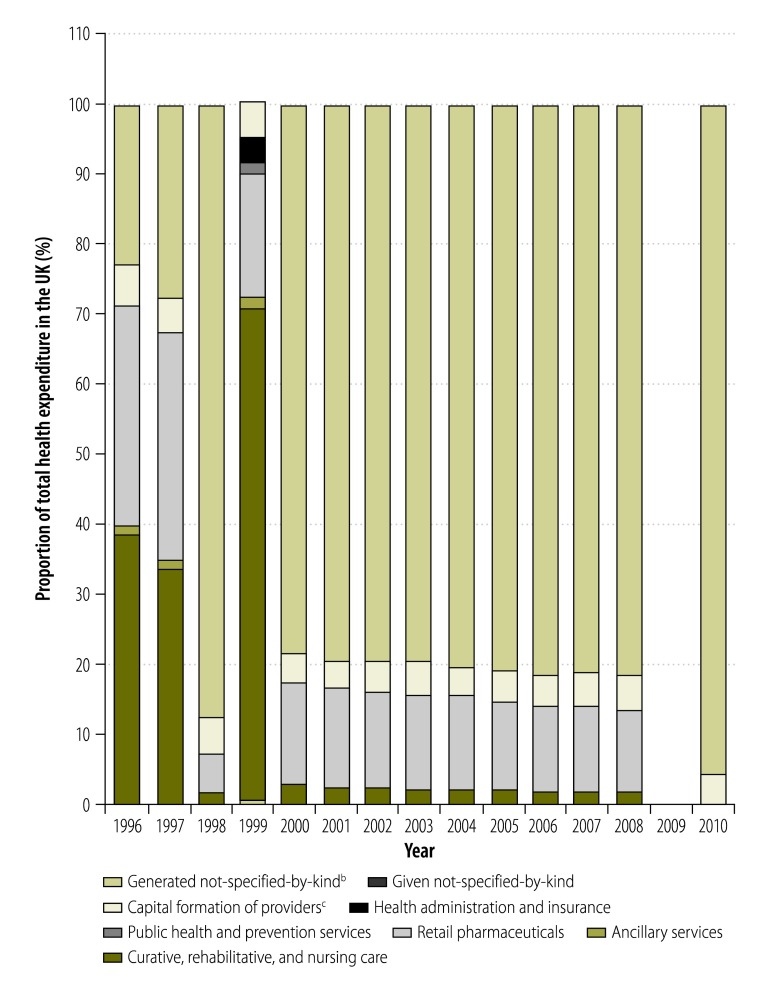
Health expenditure in the United Kingdom of Great Britain and Northern Ireland,^a^ categorized by health function, 1996–2010

^a^ The United Kingdom did not provide expenditure information by health function in 2009.

^b^ The generated not-specified-by-kind component of health expenditure was the value we generated when the sum of the reported values of individual categories within a data type (i.e. financing agent, financing source, health function or health provider) did not equal the total for that type.

^c^ Capital formation of providers refers to gross fixed capital formation (e.g. hospital buildings), inventory changes (e.g. vaccinations kept in stock) and acquisitions less disposals of valuables (e.g. artworks).^9^

On occasion, we observed jumps in expenditure for individual NHA components greater than 50% between one year and the next. For financing sources, the year-on-year change in the monetary value of expenditure by private funds exceeded 10% for 32% (71/222) of all observations. Moreover, the year-on-year changes in expenditure by both private and public funds exceeded 50% for 9% (20/222) of all observations. Large variations in the level of expenditure by different financing agents were also seen, as illustrated in Fig. 8 for Zambia. When we examined the year-on-year change in the share of total health expenditure that went to hospitals in all NHAs, we found that it was 10% or higher for 26% (134/516) of all observations. Similarly, the year-on-year change in the share that went to health administration and health insurance, as health providers, was 50% or higher for 10% (49/482) of observations. For expenditure on curative, rehabilitative and nursing care, as a health function, we found that the year-on-year change was 10% or higher for 20% (103/510) of observations. Fig. 9 shows how expenditure on health function categories changed dramatically over time in the United Kingdom of Great Britain and Northern Ireland, probably due to delayed implementation of the SHA framework.

US$: United States dollar.

^a^ All expenditure was converted into United States dollars at 2010 values.

^b^ The generated not-specified-by-kind component of health expenditure was the value we generated when the sum of the reported values of individual categories within a data type (i.e. financing agent, financing source, health function or health provider) did not equal the total for that type.

^c^ The given not-specified-by-kind component was the component of a country’s total health expenditure that did not clearly fall within one of the categories specified by the publication *A system of health accounts*.^8^

## Discussion

Our analysis of NHAs produced between 1996 and 2010 indicates that, in their current state, they are not sufficiently reliable for evaluating differences in health expenditure between countries or changes over time. Often the categories used to report expenditure were not in line with the SHA or the health satellite accounts of the system of national accounts framework. In some cases, producers of reports may have created categories that reflected types of health expenditure unique to their countries, whereas others may have categorized a large proportion of expenditure as not-specified-by-kind because of insufficient documentation. This poor adherence to established frameworks reduced the utility of NHAs as a metric of national health expenditure over this period. In addition, data series were often incomplete and changed implausibly from year to year. We observed year-on-year jumps in total expenditure over 50%. Any year-on-year change greater than 10% is extraordinarily high, particularly when it is not part of an overall increase in expenditure. It is difficult, therefore, to believe such changes were real – it is more likely they were produced by stochastic data-generation processes. Moreover, the use of not-specified-by-kind categories magnified this problem because expenditure assigned to a particular category in one year may not have been assigned to the same category in the following year.

Conversations with producers of NHAs have confirmed that poor data quality presents a systemic challenge. Although WHO and private sector organizations have provided technical assistance for the production of NHAs, more complete and plausible NHAs may have to await reforms in financial data management and tracking systems. In our analysis, the variation in data quality we observed was probably due to differences between countries in data systems, data collection methods and access to technical assistance. The high values for the not-specified-by-kind components we observed indicate that countries were generally aware of their total health expenditure but were unsure about the exact destination of the funds.

One limitation of our study is that NHA reports may have been subject to selection bias because countries with greater financial resources and technical capacities, including those assisted by development partners, would have been able to produce NHAs more frequently and to provide more complete data. In addition, we were able to collect data only from reports that were made publicly available. If a country produced an NHA but did not make it available, it could not be included in our analysis. 

Although no accounting system is perfect, there is no substitute for reliable data. Better and more extensive data will reduce errors in NHAs and minimize the value of the not-specified-by-kind component. The provision of in-depth information about not-specified-by-kind categories, categorization assumptions and data collection methods would enable researchers to compare NHA data over time and between countries. Thereafter, the information could be extended using modelling and statistical inference. Given the substantial time and resources necessary to produce NHAs, we do not recommend a periodicity for their production. Nevertheless, annual reports would provide a better basis for decision-making and would enable researchers to investigate trends in expenditure in greater depth.

Since the 1990s, national governments and development partners have invested considerable resources in producing NHA reports. Although these reports have been used to guide programmes for improving the welfare of the population, this has occurred relatively infrequently. In general, the use of NHA reports has been limited. However, as NHA data improve, we expect these reports to become increasingly useful to finance ministries for allocating resources to health systems and to health ministries for determining how best to allocate capital within the health sector. We hope that the publicly available, centralized source of NHA reports we have created will stimulate interest in existing NHA data and encourage the production of high-quality reports in the future.
